# Gas Sensor Array and Classifiers as a Means of Varroosis Detection

**DOI:** 10.3390/s20010117

**Published:** 2019-12-23

**Authors:** Andrzej Szczurek, Monika Maciejewska, Beata Bąk, Jakub Wilk, Jerzy Wilde, Maciej Siuda

**Affiliations:** 1 Faculty of Environmental Engineering, Wrocław University of Science and Technology, Wybrzeże Wyspiańskiego 27, 50-370 Wrocław, Poland; andrzej.szczurek@pwr.edu.pl; 2 Apiculture Department, Warmia and Mazury University in Olsztyn, Sloneczna 48, 10-957 Olsztyn, Poland; beata.bak@uwm.edu.pl (B.B.); teofil.wilk@uwm.edu.pl (J.W.); jerzy.wilde@uwm.edu.pl (J.W.); maciej.siuda@uwm.edu.pl (M.S.)

**Keywords:** honey bee, gas sensor, *Varroa destructor*, classification

## Abstract

The study focused on a method of detection for bee colony infestation with the *Varroa destructor* mite, based on the measurements of the chemical properties of beehive air. The efficient detection of varroosis was demonstrated. This method of detection is based on a semiconductor gas sensor array and classification module. The efficiency of detection was characterized by the true positive rate (TPR) and true negative rate (TNR). Several factors influencing the performance of the method were determined. They were: (1) the number and kind of sensors, (2) the classifier, (3) the group of bee colonies, and (4) the balance of the classification data set. Gas sensor array outperformed single sensors. It should include at least four sensors. Better results of detection were attained with a support vector machine (SVM) as compared with the k-nearest neighbors (k-NN) algorithm. The selection of bee colonies was important. TPR and TNR differed by several percent for the two examined groups of colonies. The balance of the classification data was crucial. The average classification results were, for the balanced data set: TPR = 0.93 and TNR = 0.95, and for the imbalanced data set: TP = 0.95 and FP = 0.53. The selection of bee colonies and the balance of classification data set have to be controlled in order to attain high performance of the proposed detection method.

## 1. Introduction

Honey bees have an essential role in nature that goes beyond the production of honey and beeswax. They play a vital role in the environment by pollinating both wild flowers and many agricultural crops. The honey bee population has strongly declined in recent years [1] due to a combination of multiple stresses, including diseases, pathogens and pesticides. Bees, like all animals including humans, are susceptible to viruses (e.g., deformed wing virus, Israeli acute paralysis virus, Kashmir bee virus, Black Queen cell virus and Sacbrood); bacteria (e.g., American foulbrood and European foulbrood); fungi (e.g., Chalkbrood and Stonebrood); microsporidian parasite (e.g., *Nosema apis* and *Nosema cearanae*); parasitic mites (e.g., Tracheal mites (*Acarapis woodi*), Varroa mites, and Varroa mites resistant to fluvalinate and coumaphos); and insect pests (e.g., wax moth, and small hive beetle) [2,3,4,5].

A bee colony has defense mechanisms, but sometimes they are too weak. In this situation, insects need help from a beekeeper. An essential part of beekeeping is the inspection of colonies for bee diseases. Early signs of infection normally go unnoticed; they only become apparent when infection is high. Early diagnosis of disease can prevent the losses of bee colonies across wider areas. Hence, beekeepers should be able to recognize bee diseases and parasites and to differentiate the serious diseases from less important ones. Observation is a crucial part of beekeeping [6].

Varroosis is the most destructive disease of honey bees worldwide [5]. It is caused by a *Varroa destructor*, an external parasite of adult insects and brood. This mite causes malformation of the legs and wings. Adult bees affected with varroosis have shrunken abdomens. The *Varroa destructor* mite can also act as a vector for viruses of the honey bee. Therefore, *Varroa destructor* infestations often leave honey bee colonies weakened and more susceptible to other diseases. Mites are spread to other colonies through drifting and robbing. Their high reproductive potential makes the control of varroosis a considerable challenge for beekeepers. The *Varroa destructor* mite level may be determined in a hive by monitoring natural mite fall, drone brood sampling or by using the ether roll test. Detection by close examination of the brood or testing of the adult bees is not an easy task—it is strongly subjective, time consuming and requires experience. Therefore, methods based on objective and reliable measurements would be very useful in apiculture [7,8,9,10,11]. One possibility relies on:

- the detection of specific parameters being indicators of varroosis;
- the qualitative and quantitative analysis of air inside beehive.

The gaseous mixture inside a hive presents strong temporal variability, while being unique and complex from the chemical perspective. Usually, the gas surrounding a honeybee colony is a mixture of compounds emitted by the bees themselves (e.g., pheromones, other chemicals released to repel pests and predators, metabolites, etc.), substances originating from hive stores (e.g., honey, nectar, larvae, beeswax, pollen and propolis), and volatile compounds from the materials out of which hives are constructed (wood, paint, plastic, etc.). The beehive atmosphere also contains compounds from vehicles, farms, industries and households, which are emitted in the vicinity of the hive. Sometimes bee diseases influence the indoor air surrounding a honey bee colony. For example, foulbrood is a source of a characteristic odor and experienced beekeepers with a good sense of smell can detect the disease upon opening a hive. In the case of the notorious varroa mites, it is known that they can change their surface chemicals to match the developmental stage of their hosts. The potential change of the chemical properties of the indoor air could be the basis for detection of varroosis. In the literature, it is difficult to find information about the direct influence of *Varroa destructor* mites on the chemical composition of the air surrounding bees inside the hive.

Today, there are a number of well-established methods that are capable of identifying and quantitating the specific chemical species in complex gas mixtures. Infrared analyzers, Fourier-transform infrared spectrometers (FT-IR spectrometers), or gas chromatographs with appropriate detectors (usually flame ionization detector (FID) or tuned mass spectrometer (MS) or other mass-selective detector) offer a low detection limit, good accuracy, sensitivity, and repeatability. In apiculture, these instruments have limited application, as they are prohibitively expensive and thus impractical. The measurement process in this instrumentation is time-consuming, labor-intensive and requires trained and experienced personnel. Usually, these types of equipment cannot be used in field conditions. In addition, there are not any known chemical compounds to be used as indicators of varroosis.

Shortcomings of the currently available instrumentation mean that new measurement strategies are required. These could be based on a sensor technology. According to the current IUPAC (International Union of Pure and Applied Chemistry) definition: “a chemical sensor is a device that transforms chemical information, ranging from the concentration of a specific sample component to total composition analysis, into an analytically useful signal” [12].

The method of the multicomponent gas mixture analysis is an important issue in sensor technology. The measurements can be accomplished by adopting highly selective (specific) sensors or using devices that are called and described as an electronic nose. The gas sensors produced nowadays do not allow the identification and determination of the concentration of chemicals related to varroosis. In our opinion, totally selective sensors based on key–lock interactions will not be constructed in the near future. Therefore, we propose to use the electronic nose for detection of varroosis.

The concept of the electronic nose (E-nose) was introduced in 1982 by Persaud and Dodd [13]. A formal definition of this device was published by Gardner and Bartlett in 1994. It states: “an electronic nose is an instrument which comprises an array of electronic chemical sensors with partial sensitivity and an appropriate pattern recognition system capable of recognizing simple or complex odors” [14]. The sensing elements are selected in such a way that they possibly respond to a wide range of volatiles. The sensor array function is to generate a unique signature for each gas sample. The collective response of partially selective gas sensors provides patterns, which can be translated into qualitative and quantitative information regarding the composition of a gas mixture.

Chemical sensor arrays coupled with a pattern recognition unit have been demonstrated to be highly effective tools for the discrimination of gas mixtures. Commercial E-noses began appearing in the mid-1990s. Nowadays, most applications concern foods, drinks, cosmetics, biomedical uses, pharmaceuticals, and the military industry, as well as environmental monitoring (characterization of odors). In the last two decades, the possibility of applying E-noses in agriculture has become an issue of increasing interest. The research effort is focused on sectors such as agronomy, biochemical processing, botany, cell culture, plant cultivar selections, environmental monitoring, horticulture, pesticide detection, plant physiology, and pathology. The most common applications of electronic noses in agriculture are related to:

- the monitoring of food quality [15,16,17];
- the identification of insect infestation and soil volatile fingerprints.

For example, early detection and classification of pathogenic fungal disease in post-harvest strawberry fruit by electronic nose is presented in the paper [18]. The work [19] reviews electronic noses as a fast and noninvasive approach for the diagnosis of insects and diseases that attack vegetables and fruit trees. The particular focus is on bacterial, fungal, and viral infections, and insect damage. The E-nose has successfully detected the different soil volatile fingerprints in each depth level and insect pests [20]. Lan et al. reported the identification of insect by using the E-nose [21]. Electronic noses were also proposed for the detection of:

- cotton damaged by *Nezara viridula* [22];
- insect infestation in wheat [23]; and
- insect infestation in paddy field [24].

New fields of E-nose applications are still sought for. Our work is focused on this research area. Generally speaking, electronic noses for diseases diagnosis are in their early stage of development. Many problems regarding sensor performance, sampling, and detection in field conditions, as well as scaling up measurements are challenges and have to be solved.

The aim of this study is to show that a device based on the electronic nose concept can be used for detection of varroosis infestation. It was assumed that the ability to identify early stages of insect invasion has to be ensured. It should be noted that achieving this goal is challenging as chemical indicators of varroosis are unknown. Additionally, the measurement equipment has to be inexpensive, easy to use, and work in field conditions.

The rest of the paper is structured as follows. Section 2 focuses on the materials and methods applied in the study. The device based on gas sensors is described, as well as the measurement procedure applied during beehive monitoring. The investigated object—bee colonies—are presented. Emphasis is put on the principle of colony categorization in respect to *Varroa destructor* infestation. The classification approach applied to detect varroosis based on gas sensors responses is characterized. Several issued are considered: data set balance, feature vector determination, classifier, and performance assessment metrics. Finally, the scope of statistical analysis of classification results is indicated. Section 3 presents the exemplary measurement data and the statistical analysis of the classification results. The influence of the selected factors on the performance of the varroosis detection method is examined. The factors are: (1) number and kind of sensors used as the source of the measurement data, (2) classifier, (3) choice of bee colonies, and (4) balance of the classification data set. Section 4 is dedicated to the discussion of the obtained results. Final conclusions are formulated in Section 5.

## 2. Materials and Methods

Three principal issues in our work concern: the equipment and procedure of gas sampling from the inside of the hive; the gas sensor array; and the pattern recognition unit with multivariate data processing tools.

The conditions occurring inside a hive and honey bee behavior mean that a measurement device should be located outside a beehive. Hence dynamic sampling was proposed. The insertion of the sampling probe to the hive should be minimally intrusive, in order to avoid modifying the bees’ environment. In addition, the sampling procedure and materials used to perform this operation cannot change the chemical composition of a gas sample.

The measurement potential of the sensor array depends on the number of gas sensors as well as physical and chemical properties of these devices. It is difficult to determine precisely these factors, as measurement conditions change greatly. It is difficult to take into account all circumstances. Hence, a high flexibility in choosing the sensing element is recommended. The large number of gas sensors can increase the discrimination abilities of the instrument and the value of the obtained information. This is an important advantage, as the air inside a beehive is a multicomponent gas mixture. However, too large a number of gas sensors has a negative impact on the pattern recognition process, e.g., it is a source of redundancy. This also effects the energy consumption of the instrument, as well as the size and weight. Alternative construction variants may be considered if number of sensors is small. Based on our earlier experience, we decided to use six chemical sensing elements. This number should ensure the suitability of the instrument performance in many various situations.

The commercial gas sensors available for E-nose systems can be divided, according to the working mechanism, into five different categories. Usually the principle of operation is based on the conductivity, gravimetric, electrochemical, thermal, or optical properties of the sensing material. Several criteria were taken into account at the stage of selection of gas sensors. These devices have to respond with different sensitivity not only to one target gas but to many other substances, especially volatile organic compounds and water vapour. They should present cost-effectiveness, reliability, availability, fast response, and recovery time, on-site operation, data acquisition in real time, high sensitivity, detection limit at the level of ppm. In this study, we decided to use semiconductor metal oxide sensors (MOS), because they fulfil many requirements mentioned above. Additionally, they are small and can be elements of integrated circuits. The resistance responses of semiconductor gas sensors to the tested gases are induced through the oxidation reactions of the reducing gases adsorbed on the sensing layer. Many gases may be oxidized on the surface of the semiconductor. Hence, this type of gas sensors yield nonspecific responses to multicomponent gas mixtures.

The resistance responses of individual sensors to the same gas are diverse. They are converted into electrical signals. After a collection and processing of signals, the feature vector is formed and used to solve a given classification problem. In other words, instead of qualitative and quantitative information, classes of air quality are determined. Categories are distinguished by the level of infestation was used as the reference, which was determined by other methods.

### 2.1. Measurement Device Based on Gas Sensors

**Figure 1:**
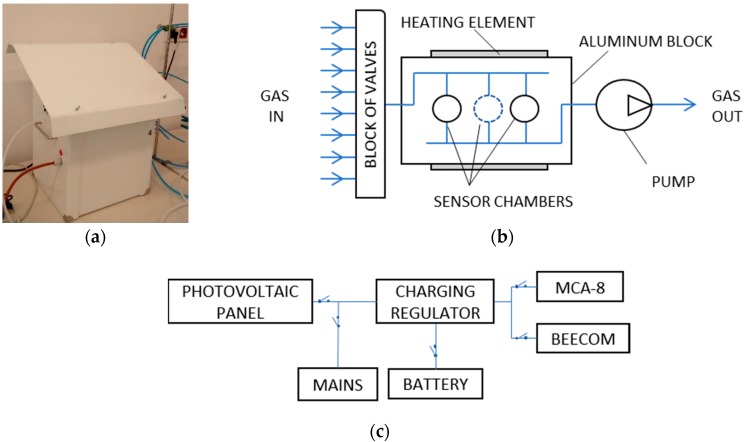
The instrument based on gas sensors. (**a**) General view, in casing; (**b**) Block diagram of multichannel recorder of gas sensor signals, model MCA-8; (**c**) Block diagram of power supply.

A prototype multisensor detector of air quality was used in the study. The construction was developed in the Laboratory of Sensor Technique and Indoor Air Quality Studies at Wroclaw University of Science and Technology, Poland. It was made by InSysPom, Poland. The detector is an autonomous, multifunctional, and programmable device based on gas sensors. It allows for continuous measurements of gas samples. Remote access to the recorded data is possible. The instrument is composed of several functional modules: (1) multichannel recorder of gas sensor signals, model MCA-8, (2) communication controller Beecom, (3) charging regulator for solar panel, Steca Solsum 6.6, (4) gel battery, HZY EV12-33, with the nominal power 36 Ah and voltage 12 V and battery level indicator, (5) photovoltaic solar panel, CL050-12P, with the nominal power 50 W, (7) AC adapters, and (6) casing. Table 1 shows the general view of the instrument (Figure 1a), the block diagram of multichannel recorder of gas sensor signals, model MCA-8 (Figure 1b) and the block diagram of power supply (Figure 1c).

The major functional unit of the device is the multichannel recorder of gas sensor signals MCA-8, see Figure 1b. It includes the following six semiconductor Taguchi gas sensors (TGS): TGS832, TGS2602, TGS823, TGS826, TGS2603, and TGS2600. They are commercially available products offered by Figaro Engineering, Japan. All used sensors respond to wide range of volatile organic compounds, as disclosed in the products information sheets [25]. Sensors that belong to series TGS2xxx have a lower detection range from 1 ppm to several dozen ppm, as compared with sensors that represent series TGS8xx. Their detection ranges are from 10 ppm to more than 1000 ppm.

Gas sensors were mounted in individual sensor chambers, made of aluminum. Chambers were located inside the aluminum block. The volume of chambers designed for TGS8xx sensors was about 12 cm^3^ and volume of chambers for TGS2xxx sensors was about 4 cm^3^.

The sensitivity of chemiresistors depends on the temperature of the gas-sensing layer. For that reason, the gas sensor heater temperature was stabilized individually for each sensor. It was based on the duty cycle of the pulse-width modulation (PWM) signal, which controls the power applied to the sensor heater. The range of this parameter was 0% to 100%, and 100% was recommended by the producer. The last setting was used as default when the instrument operates in a stand-by mode i.e., when it does not perform measurements. Additionally, the gas sensor device was equipped with the setup, which controls the temperature of the aluminum block, where sensors chambers are located. The idea was to improve sensors performance by securing constant temperature of the test gas in their surroundings. The setup consisted of the heater and the fan. Ambient air was used for cooling.

The electronic circuits for measuring sensor resistance and driving sensor heaters were fabricated according to sensors technical sheets. The applied analog-to-digital conversion (ADC) resolution was 12 bit oversampled to 16 bit successive approximation register (SAR).

The device was dedicated to operate continuously and perform measurements in the dynamic mode. A miniature diaphragm pump DP0102, manufactured by NITTO KOHKI was used to enforce the gas flow. The pump was mounted inside the MCA-8. According to technical specifications, the maximum free gas flow rate for the pump was 5 L/min. The gas flow rate in the system could be adjusted. The power applied to the pump was controlled for this purpose.

The instrument was fitted with eight gas inlet ports, which could be individually connected to gas sensors chambers by means of a set of valves. This solution allowed for an intermittent gas sampling from eight locations.

As default, the measurement data was recorded on the instrument’s secure digital (SD) card, with a temporal resolution of 1 s. Optionally, the remote data transfer could be realized using Global System for Mobile Communications (GSM) (5 s resolution).

The operation of the gas sensor device is programmable. The user has to define the following parameters: duration of gas intake through individual inlet ports, pump operation rate, as well as the power of gas sensors heaters. The program is executed from the SD card upon insertion. The instrument may be also operated in an interactive mode using a PC-based software.

Three powering options are available: main power supply, battery and photovoltaic solar panel (see Figure 1c). The last two solutions were aimed to secure the autonomous operation of the device in field conditions. The casing protected the instrument against meteorological conditions.

### 2.2. Bee Colonies

One of the established, manual methods to assess *Varroa destructor* infestation of a bee colony is the flotation method. This method was used as the reference for the method based on gas sensing. The flotation method involves the collection of a bee sample from the colony. Dead bees are shaken with a detergent or alcohol, then rinsed on a sieve [26,27]. Ultimately, the mites found in a sample of bees are counted. Their number is divided by the number of bees and expressed as the percentage. The obtained parameter represents in this work *Varroa destructor* infestation rate of a bee colony. It was assumed that the results of *Varroa destructor* infestation assessment by flotation provide a reliable indication of the advancement of bee colony disease. The results obtained by flotation were utilized to examine the possibility of determining *Varroa destructor* infestation based on semiconductor gas sensor array measurements. The evaluation of the gas sensor method was adequate to the extent that the assumption was fulfilled.

Eighteen bee colonies were qualified for the experiment. The only criterion of selection was the *Varroa destructor* infestation rate, which was determined using a flotation method. There were two groups of bee colonies, A and B. Each group included nine colonies. Three of the colonies had a *Varroa destructor* infestation rate at the level of 0%, which is below the detection limit of a flotation method. The other six colonies were infested and their infestation rates were from 1.3% to 24.76% in group A and from 1.13% to 6.50% in group B, as shown in Table 1.

The status of bee colonies was additionally characterized in terms of the number of bees, amount of brood, amount of bee food and other selected parameters. These parameters were highly variable in both groups of bee colonies. In view of the objective of this work, their role was purely descriptive. We examined the possibility of detecting *Varroa destructor* infestation irrespective of the variation of other parameters of bee colonies.

### 2.3. Measurements

The air in beehives, which were inhabited by honey bee colonies listed in Table 1, was measured using the gas sensor device. In total, eighteen beehives were examined during the study.

**Figure 2:**
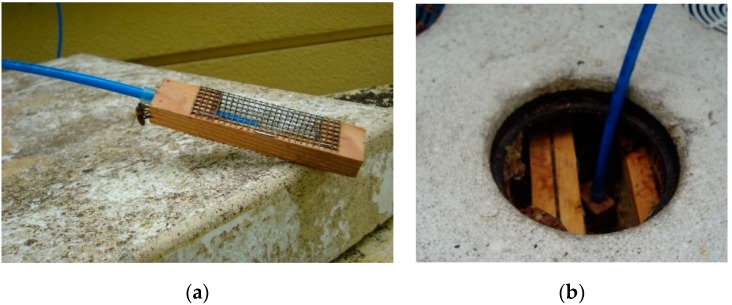
(**a**) Sampling probe; (**b**) sampling probe placement in the upper part of the beehive—top view.

During measurements, the gas sensor device was connected to bee hives by means of polyethylene (PE) tubing. One inlet port was used per one hive. The gas sampling points were located in central, upper part of beehives, between brood combs (see Figure 2b). In this location, the varroosis should be best reflected in the quality of beehive air. Sampling probes (Figure 2a) were meant to prevent clogging by bees. One additional device inlet port was dedicated to the delivery of ambient air for sensors regeneration. Ambient air was prepared for this purpose by passing it through a dedicated filter filled with charcoal. Inlet ports of the device were protected by particle filters. During measurements, the gas flow rate in the system was 0.5 L/min. Sensors were driven at a fixed voltage of 5 V and applied to heaters.

Two identical measurement devices were used in our study. An individual device was dedicated to examine one group of bee colonies, as shown in Table 1. Each group of colonies was divided in subgroups of three elements. The beehives from one subgroup were connected simultaneously to one sensor device. The three colonies were examined one by one, in sequence. Once completed, the measurement sequence was repeated. A single measurement of a bee colony consists of two phases: (1) the exposure of gas sensors to beehive air (600 s), (2) the exposure of gas sensors to the cleaned air (900 s). Multiple measurements were done for all of the bee colonies.

Field experiments were conducted in May 2019. The measurements of two groups of bee colonies were done in parallel and in the same location. Hence, the meteorological conditions encountered during the experiment were the same for both groups of bee colonies. Table 1 shows the number of measurements that successfully passed the data validation procedure. As shown, bee colonies were represented by various numbers of measurements, between 6 and 123. In most cases, several dozens of measurements per colony were available for further analysis.

The validation was done offline once per one to two days. In that process, the data associated with each single measurement was labelled as valid or invalid. Additionally, the recommendations were made to adjust the measurement set-up, if needed. Data losses were mostly associated with unfavorable meteorological conditions. The experiment was run in the early spring of 2019. Unfortunately, at night temperatures oftentimes dropped down several degrees. Water vapor condensation inside PE tubing was observed as well as the moisturization of the gas lines. Measurements had to be interrupted in order to dismantle the measurement setup and dry it.

The data validation was heuristic. Our domain-specific knowledge and measurement experience was utilized to separate valid and invalid data. Two major criteria were used: (1) The characteristic shape of gas sensor signal recorded during single measurement had to be preserved. (2) The similarity to the sensor signals recorded during repeated measurements of the same beehive had to be maintained.

### 2.4. Classification

#### 2.4.1. Categories of Bee Colonies

This work is focused on the detection of *Varroa destructor* infestation of bee colonies based on the classification of responses of the gas sensor array to beehive air. Two categories of bee colonies were distinguished: ‘not infested’ and ‘infested’. The colonies assignment was based on the infestation rate, which was estimated using the flotation method. We arbitrarily assumed that in the category ‘not infested’, the infestation rate of bee colonies was 0%, while in the category ‘infested’, the infestation level was greater than 0%. In practice, the range from 1.3% to 24.76% was taken for group A and from 1.16% to 6.50% for group B. The classification was studied separately for the two groups of bee colonies.

#### 2.4.2. Data Preprocessing

The gas sensor signal recorded during a single measurement of the bee colony consisted of two parts, which corresponded to two phases of the measurement. When exposed to beehive air, gas sensor responses increased and afterwards they reached the quasi-steady state, representing the equilibrium with beehive air. When gas sensors were exposed to the regeneration air, their responses decreased, but the initial pre-exposure value was not always retrieved. In order to establish the common baseline for gas sensor signals recorded during different measurements, differential baseline correction was applied. This consisted of subtracting a predefined value from the signal. The value was the average gas sensor response during the last 30 s of regeneration phase, before the exposure to the gas sample.

#### 2.4.3. Feature and Feature Vector

A feature was defined as the response of the individual gas sensor, associated with the selected time of exposure to beehive air. The following times of exposure were considered: 5 s, 15 s, 25 s, …, 115 s. These 12 time points represented the first two minutes of the gas sensors exposure to beehive air. If the discriminative potential of this part of signal is confirmed, varroosis detection may be based on a relatively short measurement.

The feature vector was composed of features selected from the responses of various semiconductor gas sensors included in gas sensor array. Various feature vectors were considered in the study. We used vectors composed of features selected from:

- The signal of an individual sensor; 1 × 12 features in one vector; six feature vectors (six single sensors);
- The signals of two sensors; 2 × 12 features in one vector; 15 feature vectors (15 combinations of two sensors out of six);
- The signals of three sensors; 3 × 12 features in one vector; 20 feature vectors (20 combinations of three sensors out of six);
- The signals of four sensors; 4 × 12 features in one vector; 15 feature vectors; (15 combinations of four sensors out of six);
- The signals of five sensors; 5 × 12 features in one vector; 6 feature vectors; (six combinations of five sensors out of six);
- The signals of six sensors; 6 × 12 features in one vector; one feature vector; (one set of six sensors).

An individual classifier was built for each feature vector.

#### 2.4.4. Oversampling

The measurement data collected for two groups of bee colonies, A and B, were imbalanced in terms of category representation. Not infested bee colonies were represented by 45.63% of measurements in the data associated with group A, and only by 27.83% of measurements in the data set associated with group B. The problem of imbalanced measurement data sets may be inherent in the considered application. Therefore, we examined the importance of this factor for the performance of the varroosis detection method. Imbalanced and balanced data sets were used as inputs of classification models. Balanced data sets corresponding to imbalanced data sets were formed of measurements associated with bee colony groups A and B, respectively. The oversampling of the underrepresented category was applied to attain this goal [28]. In the balanced data set, the same number of data vectors represented each category. To reach the balance, the number of data vectors in the ‘not infested’ category was increased while the number of data vectors in the category ‘infested’ has not been changed. The oversampling procedure consisted of a random drawing with a replacement under the assumption of uniform probability distribution.

#### 2.4.5. Classifier

Two classification methods were used: the k-nearest neighbors algorithm (k-NN) and support-vector machine (SVM). The k-NN algorithm [29,30] was chosen for its simplicity. It is easy to program and it may be successfully embedded in the data processing unit of the measurement device. K-NN is a nonparametric, nonlinear and distance-based method. The class assignment is based on the smallest distance between the test vector and training vectors, which represent various classes. Multiple distance measures may be utilized; however, in this study Euclidean distance was applied. In the case of k-NN, there is no strict learning phase. Training vectors are retained in the memory and they are called each time a new vector is classified. The test vector is assigned to the class which most frequently occurs among k training vectors, nearest to it. k is the only parameter of the method—it is chosen arbitrarily or by the trial and error method, which allows the avoidance of the lengthy process of classifier optimization. We chose k = 3. Highly nonlinear decision boundaries may be represented using the k-NN technique.

SVM was chosen as a presumably better classifier [31]. The algorithm realizes a very efficient supervised classification. Namely a set of data vectors with known class assignment is needed in order to train a classification model, which can later be used for classification of test data vectors. At the training stage, the SVM constructs a hyperplane or set of hyperplanes in a high- or infinite-dimensional space, which separate distinct classes of data. The original data, which has a defined dimensionality is mapped into this high- or infinite-dimensional space in order to attain, or at least approach linear separability of classes. The constructed hyperplane is meant to have the largest functional margin i.e., the largest possible distance to the nearest training-data point of any class. This assures relatively high generalization. In principle, SVM is dedicated to solve two-class classification problems.

#### 2.4.6. Classification Performance Indicators

The detection of bee colony infestation using the gas sensor array was addressed as the two-class classification problem. The evaluation of classification performance was based on a confusion matrix, as shown in Table 2. If classification serves the medical diagnostics, the elements of the confusion matrix have an established interpretation. Traditionally, negative (N) means the true lack of the disease, and positive (P) represents its true presence. As the detection of bee colony infestation with the *Varroa destructor* mite is the diagnostic task, the relevant terminology was adopted in this work. Negative (N) refers to ‘not infested’ bee colonies and Positive (P) refers to the ‘infested’ colonies.

The performance of classification was examined using true positive rate (TPR = TP/TP + FN) i.e., sensitivity, true negative rate (TNR = TN/TN + FP) i.e., specificity and false positive rate (FPR = 1 − TNR). TPR and TNR allow the identification of the correct class assignments with reference to the true categories. The information about both rates is very important when the costs of false positive detections and false negative detections are unspecified.

In this work, equal costs of false positive and false negative detections were assumed. However, such assumptions may be questioned. In fact, when the true positive rate decreases, an increasing number of infested bee colonies are classified as healthy ones. Classification of ill colonies as healthy is unfavored because the colonies which shall undergo medical treatment may be left without it or receive too small of doses. On the other hand, when the false positive rate increases, the increasing number of healthy bee colonies are classified as infested with *Varroa destructor*. The classification of healthy colonies as ill ones may generate excessive costs, due to the consumption of unnecessary medical treatment. This also raises the issue of the consequences of excessive exposure of a bee colony to medicines, including losses of revenue due to setting the productive colony aside for treatment.

Ten-fold cross-validation was applied to examine the performance of classification. The validation procedure was repeated 30 times for each classification model.

### 2.5. Statistical Analysis

The results of classification were statistically analyzed in respect of factors which could have an influence on the classification performance, and ultimately on the performance of the varroosis detection method. The factors were:

- classifier (SVM and k-NN);
- group of bee colonies for which the measurements were performed and then applied for classification model training (group A and group B);
- balance of the classification data set (balance: equal representation of categories, and imbalance: representation of categories as in the validated measurement data sets);
- combination of gas sensors which was used as the source of data for classification (all possible subsets of six sensors included in the gas sensor array were considered, we grouped them by size, i.e., one, two, three, four, five, and six-element combinations).

The classification option was defined by the particular combination of these factors.

The examination of factors, which influence the classification performance, was based on the analysis of TPR and TNR, which were chosen as the indicators of classification performance. TPR and TNR, computed for different classification options, were compared. The applied statistical tools were the one-way analysis of variance and multiple comparisons, based on the Tukey–Kramer test.

MathWorks software was used for data preprocessing and analysis. In particular, Statistics and Machine Learning Toolbox was applied to realize the classification and to examine its performance.

## 3. Results

**Figure 3:**
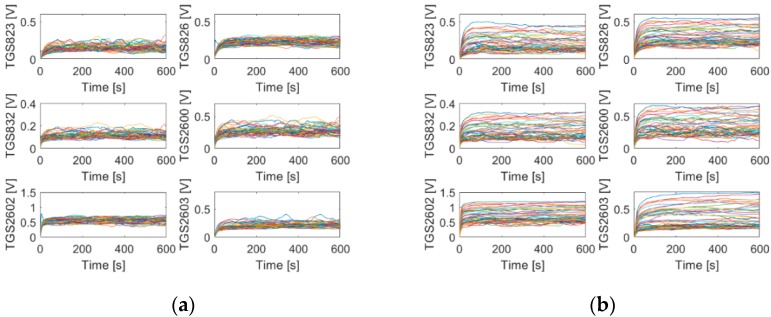
Gas sensor signals recorded during measurements of beehives occupied by two exemplary bee colonies. (**a**) Colony A1—not infested by *Varroa destructor* (see Table 1); (**b**) colony A2—infested at the rate of 25.76% (see Table 1). Differential baseline correction was applied.

Figure 3 presents signals recorded by gas sensor array during measurements of air in two beehives occupied by exemplary colonies of honey bees. One colony was not infested by *Varroa destructor*—infestation rate 0% (colony A1, Table 1), and the other was heavily infested—infestation rate of 25.76% (colony A2, Table 1).

As shown in Figure 3, gas sensor signals recorded during measurements of air in the beehive occupied by the infested bee colony (Figure 3b) were higher, as compared with the not infested colony (Figure 3a). However, there was also an overlap between results of measurements of two bee colonies that were done with the same sensor. All individual panels in Figure 3 reveal that gas sensor signals recorded during multiple measurements of one beehive displayed a considerable spread. The presented example is representative and it reflects the regularities that were generally observed in our measurements.

Table 3 displays TPR and TNR. They represent the performance of classification of bee colonies infestation by *Varroa destructor*, based on gas sensor array measurements. The considered classification options were indicated by three elements: type of classifier (SVM and k-NN), group of bee colonies, which were monitored and provided data for classifier training (A and B), and balance in the classification data set (balance and imbalance). For a particular classification option, we averaged TPRs and TNRs obtained using different combinations of sensors as the source of classification data. As shown in Table 3, the best results of classification were obtained using SVM and balanced data sets. This result is indicated by the highest values of TPR and TNR. It refers to both groups of bee colonies, A and B.

Table 4 displays *p*-values resulting from of the analysis of variance, which examined the statistical significance of the kind of classifier as a factor that influences classification performance. As shown, this factor significantly influenced TPR as well as TNR and its impact on TPR was stronger. The statistical significance of the kind of classifier was confirmed at the significance level α = 0.01. The comparison of Table 4 and Table 3 indicates the statistically significant domination of SVM.

Table 5 displays *p*-values resulting from of the analysis of variance, which examined the statistical significance of the balance in classification data set as a factor that influences the classification performance. As shown, this factor significantly influenced TPR as well as TNR and its impact on TNR was stronger. The statistical significance of the input data set balance was confirmed at the significance level α = 0.01. The comparison of Table 5 and Table 3 indicates the statistically significant domination of the balanced data sets.

Table 6 displays *p*-values resulting from of the analysis of variance, which examined the statistical significance of the selection of group of bee colonies, for which the measurement data was collected, as a factor that influences classification performance. As shown, this factor significantly influenced TPR as well as TNR, except for TNR in the case of imbalanced data sets. However, in this last case, the TNR were unacceptably small (see Table 3). The statistical significance of the selection of group of bee colonies was confirmed at the significance level α = 0.01. The comparison of Table 6 and Table 3 indicates that statistically significantly better results were attained for group B of bee colonies.

**Figure 4:**
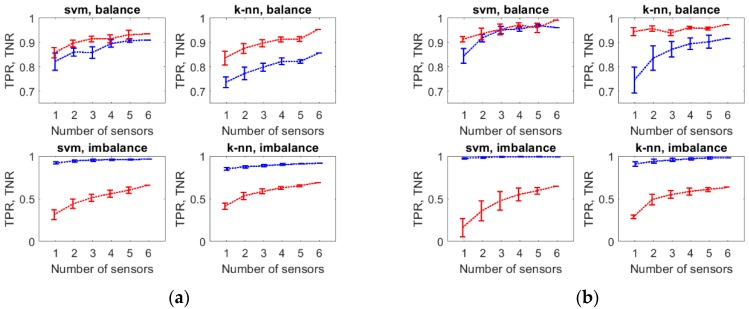
(**a**) True positive rate (TPR) and true negative rate (TNR) for classification options using the measurement data collected for group A of bee colonies; (**b**) TPR and TNR for classification options using the measurement data collected for group B of bee colonies. A mean ± standard deviation was shown for each group of models utilizing various combinations of sensors as the source of input data, while the size of combination was fixed. TPR is displayed with a blue line and TNR is displayed with a red line.

We examined the combination of sensors, which delivers the measurement data for classification, as a factor that influences the performance of varroosis detection. Figure 4 presents TPR and TNR for various options of classification with reference to bee colony group A (Figure 4a) and group B (Figure 4b). Options utilized data provided by different combinations of sensors, while the size of combination was fixed. The plots in Figure 4 demonstrate that the performance of classification was related to the number of sensors included in the combination. In general, both TPR and TNR increased with the increasing number of sensors, which is synonymous with the classification performance improvement.

Table 7 displays *p*-values obtained from multiple comparisons, which examined the statistical significance of the number of sensors as a factor that influences classification performance. The results show that in the case of one sensor, the performance was significantly different (lower) than when using two, three, four, or five sensors. In the case of two sensors, the performance was significantly different (lower), than when using three, four, or five sensors. Considering three sensors, the performance was significantly different (lower) than in the case of four or five sensors for group A of bee colonies. For group B, the differences were not statistically significant at the significance level α = 0.01. The classification performance attained when using four or five sensors was not significantly different for both groups of bee colonies. There is only one combination of six sensors (full array), so it was excluded from multiple comparisons.

**Figure 5:**
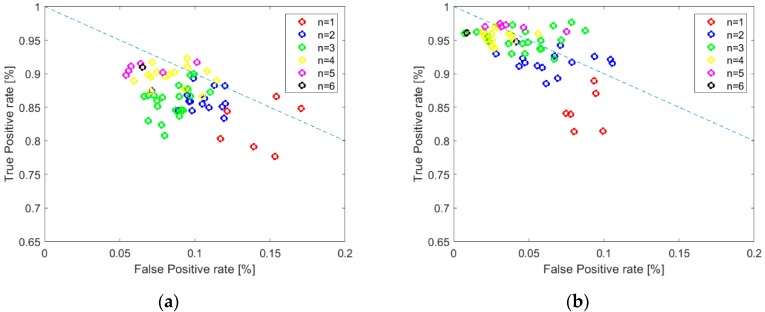
Relationship between the true positive rate and the false positive rate for classification of bee colony infestation by *Varroa destructor* based on gas sensor array responses, using support-vector machine and balanced data sets. Various combinations of sensors were considered, their size was n. (**a**) Results based on the measurement data collected for bee colony group A. (**b**) Results based on the measurement data collected for bee colony group B.

The detailed results of classification obtained with the best classifier (SVM), which operated on the balanced data sets are shown in Figure 5, with reference to bee colonies group A (Figure 5a) and group B (Figure 5b). The mutual relationship of TPR and FPR (1-TNR) was displayed for all combinations of sensors employed as the sources of the classification data (including entire sensor array). The plots allow us to see the distribution of TPRs, as well as FPRs, for sets of sensors that have the same size. Performance overlaps between these sets are well demonstrated. As shown in Figure 5a, as well as in Figure 5b, the complete gas sensor array offered very high classification performance. Although better results could be attained using smaller sensor sets, the composition of the best performing sensor set was case dependent.

In the coordinates of FPR and TPR, the best classifying solutions are located on the straight line between (FPR = 1, TPR = 0) and (FPR = 0, TPR = 1), close to the last point, if costs of false positive and false negative detections are equal. This line indicates the symmetry of FPRs and FNRs. In the case of classification utilizing SVM and balanced data sets, the symmetry between these rates was very high, see Figure 5. The comparison of plots in Figure 5a,b shows that the detection method performed better in the case of measurement data collected for bee colonies from group B (Figure 5b).

## 4. Discussion

This study was dedicated to a new method of varroosis detection. The presented method utilizes a semiconductor gas sensor array and classifier. This method is based on the measurement of chemical properties of beehive air. We assumed that gas sensors are capable of detecting beehive air quality changes, which could be attributed to the occurrence of the disease. The proposed approach is new and original, and was first announced in [10]. So far, the development of instrumental detection of varroosis has been mainly based on optical methods [8].

The performance of a method utilizing gas sensors could be influenced by multiple factors that are associated with the measurement technique itself, classification approach, as well as the studied object. In this work, we focused on: (1) the number and kind of sensors used as the basis of detection, (2) the kind of classifier, (3) the balance of the classification data set, and (4) the group of bee colonies for which the measurement data was collected.

We found that the composition of the sensor set that delivers the data used for varroosis detection is a factor that influences the performance of the method in a statistically significant manner (see Table 7, as well as Figure 4 and Figure 5). The obtained results provide a strong argument in favor of the application of the gas sensor array as compared with single gas sensors for the detection of bee colony infestation with *Varroa destructor*. Although sensor arrays generally perform better than single sensors, the rate of improvement is case-specific and it may not be attractive in a particular case. Examples of efficient class separation, based on measurements done with single sensors, have been announced [32]. In this study, the performance of single sensors was very low when compared with multisensor combinations, as shown in Figure 4a,b and Figure 5a,b. In general, the use of a greater number of sensors offered better performance of varroosis detection. Based on the obtained results, the complete gas sensor array, composed of six TGS sensors, was not always the best source of information, see Figure 5a,b. However, in our opinion, the number of sensors, which are physically mounted in the array, should not be reduced. The results of the statistical analysis showed that at least four sensors have to be included in the array for attaining high performance, which is significantly different than in case of one, two, or three sensors (Table 7). However, individual combinations of five or four sensors offered different performances. Moreover, the performance of a particular sensor set was dependent on the monitored group of bee colonies, compare Figure 5a,b. In this situation, the reduction of number of sensors in the array would not be recommended. Instead, multiple classifiers may be developed based on various sensor subsets and included in a committee of classifiers. This proposal deserves further consideration.

Based on classification performance indicators, the comparison of two classifiers, SVM and k-NN, revealed the domination of SVM, see Table 3 and Table 4. This result was expected. SVM is widely announced as a very effective classifier in many applications. Major performance differences were observed in terms of TPRs, while when considering TNRs the two classifiers were similar (see Table 1). Namely, SVM more effectively recognized healthy colonies as healthy. If the emphasis was on the detection of infested colonies as infested (TPR), i.e., assuming asymmetry in false classification costs, k-NN could still be considered as an option. Importantly, a balanced composition of the classification data set has to be assured. This result is very important in view of the problem of choosing a classifier for the direct implementation in the measurement device.

The results of the performed analysis indicated that the balance in the classification data set is a statically significant factor, influencing the performance of varroosis detection (see Table 3 and Table 5). In the examined scenario, the measurement data applied for training classification models was acquired during field experiments. Hence, to assure the balanced class representation, the number of bee colonies representing different categories should be the same and the number of measurements performed for each class, should be equal as well. However, field conditions are predictable to a limited extent. Hence, even if the measurement experiment is properly designed, class representation may be imbalanced. This work shows that in such cases, the balance should be reinstated, for example using oversampling.

Based on our study, the set of measurements results, which were used for training the classification model, had an influence on the performance of the varroosis detection method. Namely, TPRs and TNRs were significantly different when the classification was based on measurements of beehives occupied by colonies from group A and B (see Table 3 and Table 6). In our experiments, the measurement results were influenced by: the selection of bee colonies, the organization and realization of measurements as well as the measurement device. The least important factor was the measurement device, as twin instruments were used in the study. Their construction was identical and they realized the same measurement procedure. The organization of measurements could be meaningful, in particular, the number and temporal distribution of multiple measurements of individual colonies. We currently examine the importance of this factor in more detail. However, it is most likely that major differences in the performance of the detection method were related to the selection of bee colonies, which represented individual categories. In this experiment, colonies were not standardized. Another study is planned to evaluate the role of this factor in a systematic manner.

## 5. Conclusions

The results of the study demonstrated that the approach based on gas sensor array measurements and classification may be applied to detect varroosis, a common disease of honeybees. However, certain conditions had to be fulfilled in order to assure high performance of the method. Our analysis showed that, a single semiconductor gas sensor was insufficient as the source of the informative measurement data. The detection of the disease has to be based on the responses of a gas sensor array, which consists of several semiconductor gas sensors. The performance of the method was heavily dependent on the applied classifier. Based on the comparative analysis, which included SVM and k-NN, the former algorithm was preferred. SVM achieved the highest true positive rate (0.93) and highest true negative rate (0.95). The obtained results also indicated the importance of the balanced representation of categories in the data set, which was used to train the classification model. The use of imbalanced data set resulted in a small, basically unacceptable TNR. We also demonstrated that the performance of varroosis detection may differ in a statistically significant manner between two exemplary groups of bee colonies. This result emphasizes the fact that the adequate selection of bee colonies as well as the proper design of the measurement experiment are crucial to attain a reliable classifications model and realistic estimation of the performance of the proposed method of varroosis detection.

## Figures and Tables

**Table 1:** Bee colonies examined in the study, their *Varroa destructor* infestation rates and the number of valid measurement results. Two identical measurement devices were used, one instrument per one group of bee colonies (A and B).

	Bee Colonies Group A			Bee Colonies Group B	
ID	*Varroa Destructor* Infestation Rate (%)	Number of Valid Measurement Results	ID	*Varroa Destructor* Infestation Rate (%)	Number of Valid Measurement Results
1A	24.76	45	1B	2.36	29
2A	0.00	47	2B	0.00	6
3A	3.45	6	3B	6.50	23
4A	2.20	123	4B	2.04	22
5A	0.00	33	5B	0.00	19
6A	1.30	31	6B	1.16	14
7A	2.21	70	7B	3.40	50
8A	0.00	66	8B	0.00	39
9A	2.00	45	9B	1.13	92

**Table 2:** Confusion matrix [28].

	Predicted Negative	Predicted Positive
Actual Negative	True negative (TN)	False positive (FP)
Actual Positive	False negative (FN)	True positive (TP)

**Table 3:** **True positive rate** (TPR) and true negative rate (TNR) for various options of classification. For each option of classification, we averaged the results obtained using different combinations of sensors as the source of input data.

Option of Classification	Group A of Bee Colonies		Group B of Bee Colonies	
	Mean TPR	Mean TNR	Mean TPR	Mean TNR
SVM, balanced	0.8701	0.9069	0.9362	0.9506
k-NN, balanced	0.7953	0.8924	0.8604	0.9513
SVM, imbalanced	0.9505	0.4993	0.9889	0.4507
k-NN, imbalanced	0.8886	0.5756	0.9543	0.5274

**Table 4:** *p*-values resulting from of the analysis of variance, which examined the significance of the kind of classifier as a factor that influences classification performance.

Compared Options of Classification	Group A of Bee Colonies		Group B of Bee Colonies	
	*p*-Value for TPR	*p*-Value for TNR	*p*-Value for TPR	*p*-Value for TNR
SVM, balanced vs. k-NN, balanced	6.71 × 10^−25^	2.67 × 10^−3^	6.71 × 10^−25^	2.67 × 10^−3^
SVM, imbalanced vs. k-NN, imbalanced	2.31 × 10^−38^	8.35 × 10^−7^	2.31 × 10^−38^	8.35 × 10^−7^

**Table 5:** *p*-values resulting from of the analysis of variance, which examined the significance of the balance of classification data set as a factor that influences classification performance.

Compared Options of Classification	Group A of Bee Colonies		Group B of Bee Colonies	
	*p*-Value for TPR	*p*-Value for TNR	*p*-Value for TPR	*p*-Value for TNR
SVM, balanced vs. SVM, imbalanced	2.17 × 10^−36^	5.17 × 10^−65^	2.17 × 10^−36^	5.17 × 10^−65^
k-NN, balanced vs. k-NN, imbalanced	8.65 × 10^−39^	4.83 × 10^−62^	8.65 × 10^−39^	4.83 × 10^−62^

**Table 6:** *p*-values resulting from of the analysis of variance, which examined the significance of the group of bee colonies, for which the measurement data was collected as a factor that influences classification performance.

Option of Classification Compared Between Groups of Bee Colonies	*p*-Value for TPR	*p*-Value for TNR
SVM, balanced	2.47 × 10^−19^	2.87 × 10^−17^
k-NN, balanced	1.89 × 10^−12^	1.11 × 10^−28^
SVM, imbalanced	4.67 × 10^−35^	3.53 × 10^−2^
k-NN, imbalanced	4.58 × 10^−31^	2.06 × 10^−3^

**Table 7:** *p*-values resulting from multiple comparisons, which examined the significance of the number of sensors used as sources of classification data, as a factor that influences classification performance.

Compared Numbers of Sensors	Group A of Bee Colonies		Group B of Bee Colonies	
	*p*-Value for TPR	*p*-Value for TNR	*p*-Value for TPR	*p*-Value for TNR
1 vs. 2	0.002	0.000	0.000	0.130
1 vs. 3	0.003	0.000	0.000	0.000
1 vs. 4	0.000	0.000	0.000	0.000
1 vs. 5	0.000	0.000	0.000	0.001
2 vs. 3	0.997	0.005	0.000	0.052
2 vs. 4	0.000	0.010	0.000	0.000
2 vs. 5	0.000	0.000	0.000	0.060
3 vs. 4	0.000	1.000	0.927	0.059
3 vs. 5	0.000	0.146	0.099	0.932
4 vs. 5	0.770	0.170	0.348	0.761
